# Quercetin Attenuates Oxidative Stress and Apoptosis in Brain Tissue of APP/PS1 Double Transgenic AD Mice by Regulating Keap1/Nrf2/HO-1 Pathway to Improve Cognitive Impairment

**DOI:** 10.1155/2024/5698119

**Published:** 2024-08-28

**Authors:** Meijia Cheng, Changbin Yuan, Yetao Ju, Yongming Liu, Baorui Shi, Yali Yang, Sian Jin, Xiaoming He, Li Zhang, Dongyu Min

**Affiliations:** ^1^ Affiliated Hospital of Liaoning University of Traditional Chinese Medicine Experimental Center of Traditional Chinese Medicine, Shenyang 110032, China; ^2^ Liaoning University of Traditional Chinese Medicine, Shenyang 110847, China; ^3^ Key Laboratory of Ministry of Education for TCM Viscera-State Theory and Applications Liaoning University of Traditional Chinese Medicine, Shenyang 110847, China

**Keywords:** *β*-amyloid, acetylcholinesterase, Alzheimer's disease, apoptosis, neurodegeneration, oxidative stress, quercetin

## Abstract

**Objective:** The objective of the study is to investigate whether quercetin ameliorates Alzheimer's disease (AD)–like pathology in APP/PS1 double transgenic mice and its hypothesized mechanism, contributing to the comprehension of AD pathogenesis.

**Methods:** A total of 30 APP/PS1 transgenic mice were randomized into model group (APP/PS1), quercetin group (APP/PS1+Q), and donepezil hydrochloride group (APP/PS1+DON). Simultaneously, there were 10 C57 mice of the same age served as a control group. Three months posttreatment, the effects of quercetin on AD mice were evaluated using the Morris water maze (MWM) test, Y maze experiment, immunohistochemistry, immunofluorescence, and western blotting.

**Results:** Results from the water maze and Y maze indicated that quercetin significantly improved cognitive impairment in APP/PS1 transgenic AD mice. Additionally, serum enzyme-linked immunosorbent assay (ELISA) results demonstrated that quercetin elevated MDA, superoxide dismutase (SOD), CAT, GSH, acetylcholine (ACh), and acetylcholinesterase (AChE) levels in AD mice. Hematoxylin-eosin (HE) staining, Nissl staining, and hippocampal tissue thioflavine staining revealed that quercetin reduced neuronal damage and A*β* protein accumulation in AD mice. Western blot validated protein expression in the Kelch-like ECH-associated protein 1 (Keap1)/nuclear factor erythroid 2–related factor 2 (Nrf2)/HO-1 pathway associated with oxidative stress and apoptosis, confirming quercetin's potential molecular mechanism of enhancing AD mouse cognition. Furthermore, western blot findings indicate that quercetin significantly alters protein expression in the Keap1/Nrf2/HO-1 pathway. Moreover, molecular docking analysis suggests that Keap1, NQO1, HO-1, caspase-3, Bcl-2, and Bax proteins in the Keap1/Nrf2/HO-1 pathway may be potential regulatory targets of quercetin. These findings will provide a molecular basis for quercetin's clinical application in AD treatment.

**Conclusion:** Quercetin can improve cognitive impairment and AD-like pathology in APP/PS1 double transgenic mice, potentially related to quercetin's activation of the Keap1/Nrf2/HO-1 pathway and reduction of cell apoptosis.

## 1. Introduction

Alzheimer's disease (AD) is a neurodegenerative disease showing extensive early amyloid *β* plaque deposition and intracellular neurofibrillary tangles (NFTs) with hyperphosphorylated tau protein. Losing synapses and neurons, neurodegeneration, and cognitive decline in AD patients lead to dementia [1–3]. Nearly 35.6 million people worldwide have been infected with AD, with an increase of 4.6 million cases each year, and its prevalence increases with age. Since the age of 60, the incidence doubles every 5 years [4]. Scholars at home and abroad have undertaken significant research on AD. Since the pathogenesis of AD is complex, AD treatment becomes a challenging task.

Oxidative stress induced by the accumulation of reactive oxygen species (ROS) in cells is essential in the pathogenesis of various neurodegenerative diseases [5]. Oxidative stress has a central role in the pathogenesis of AD, which participates in AD development by enhancing A*β* production and tau hyperphosphorylation. This suggests that antioxidants could become a potential treatment for AD. There was evidence that the Kelch-like ECH-associated protein 1 (Keap1)/nuclear factor erythroid 2–related factor 2 (Nrf2) pathway is vital in maintaining the physiological balance of oxidative stress parameters and cell integrity [6]. Changes in Keap1, closely associated with Nrf2, separates Nrf2 from Keap1 and translocates from the cytoplasm to the nucleus. This activates the antioxidant gene transcription by binding to antioxidant reactive kinases, including NQO1, GSH, superoxide dismutase (SOD), and HO-1 [7, 8]. Nrf2 is an oxidative pathway protein balancing intracellular ROS and protecting cells from death caused by free radical stress [9], linked with AD-mediated cognitive decline [10]. Previous studies have revealed that Nrf2 expression within the brain decreases in the AD mouse model, and the reduction of Nrf2 accelerates cognitive impairment. Activating Nrf2 protects against harmful stress by elevating an antioxidant defense pathway and improves the cognitive impairment of AD model mice [11]. Therefore, Nrf2 could be a new therapeutic target against AD.

Quercetin is a flavonoid member extracted mainly from vegetables and fruits [12]. Quercetin has various pharmacological properties as a popular bioflavonoid, including anti-inflammation [13], antioxidation [14], immune regulation, antiamyloid production, and neuroprotective activity [3]. Several studies have reported the neuroprotective effects of quercetin within in vitro and in vivo models of neurodegenerative diseases, including AD [15], ischemia, Parkinson's disease [16], and Huntington's chorea [17]. Quercetin administration reduced the production of A*β* plaque, tau pathology, and microglia proliferation within the hippocampus of AD model mice. This improves memory and cognitive and emotional dysfunction in mice [18]. In addition, Olayinka et al. observed that quercetin alleviates memory impairment in scopolamine mice by inhibiting neuroinflammation and neurodegeneration [19]. After quercetin treatment in 3xTgAD mice, AD was reversed because tau hyperphosphorylation and A *β*1–40 and A *β* 1–42 deposition levels were inhibited in the brain [20]. The APP/PS1 transgenic mice simultaneously express mutant human amyloid beta precursor protein SWE (APPswe) and mutant presenilin-1 (PS1DeltaE9). These mice exhibit more significant pathological symptoms and can simulate the development of AD more effectively compared to other models [21, 22]. Consequently, APP/PS1 mice have been widely used in AD research, chosen as the AD model for this study. Han et al.'s study revealed that *Inonotus obliquus* polysaccharide could thwart AD by modulating Nrf2 signaling [23]. Furthermore, studies have shown that activation of the Keap1/Nrf2/HO-1 pathway in the APP/PS1 transgenic AD mouse could lessen oxidative damage and enhance cognitive function, demonstrating its crucial role in the onset of AD [24]. Moreover, quercetin, an essential small molecule antioxidant, has demonstrated a lowering effect on neuronal oxidative stress levels in Feng's research, primarily through regulating the Nrf2/HO-1 pathway to suppress ROS production [25, 26]. The study is aimed at determining whether the protective effect of quercetin on neurons is associated with oxidative stress and apoptosis regulated by its Keap1/Nrf2/HO-1 pathway. This study could clarify the potential molecular mechanism of quercetin.

## 2. Materials and Methods

### 2.1. Reagents and Antibody

Reagents and antibody used are as follows: quercetin (TargetMol, T2174), donepezil hydrochloride (DON) (Anlisin, H20050978), acetylcholine (ACh) (YJ401805), acetylcholinesterase (AChE) (YJ037240), ROS (JLC164810), SOD (ML641059), MDA (YJ077384), glutathione (GSH-Px) (YJ097316), enzyme-linked immunosorbent assay (ELISA) Kit (enzyme-linked, Shanghai, China), RIPA lysate (Biyuntian, P0013B), protease inhibitor (Bausch, AR1179), SDS-PAGE protein sample buffer (Bausch, AR1112-10), HO-1 (Abcam, ab189491), Nrf2 (Abcam, ab89443), Keap1 (Abcam, ab89443), NQO1 (Abcam, ab189491), Bax (Abcam, ab32503), Bcl-2 (Abcam, ab196495), caspase-3 (Abcam, ab184787), HRP (Abcam, ab205718) A*β* (NBP2,13075SS), and *β*-actin (Cell Signaling,8H10D10).

### 2.2. Animals

**Figure 1:**
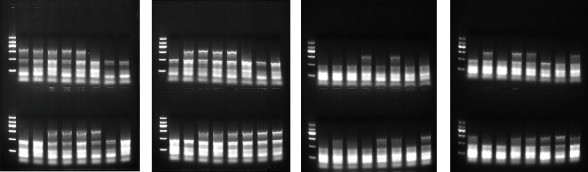
APP/PS1 gene identification.

**Figure 2:**
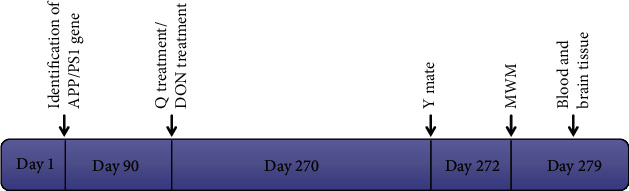
Schematic diagram depicting the experimental animal drug administration and experimental time.

The animals were kept in environmentally controlled vivariums (temperature: 20 ± 2°C; humidity: 60% ± 5%; 12 h dark/light cycle). The animals were fed standard laboratory chow with free water access. This study was conducted based on the recommendations provided by the “Guide for the Care and Use of Laboratory Animals” Laboratory Animal Care and Use Committee of Liaoning University of Traditional Chinese Medicine. The Animal Ethics Committee of the Affiliated Hospital of Liaoning University of Traditional Chinese Medicine (2022CS(DW)-010-01) approved the study protocol. PCR analysis of toes DNA helped perform the genetic identification as APP/PS1 genotype. We used 3-month-old APP/PS1 double transgenic and age-matched C57BL/6 mice as controls. The identification of APP/PS1 double transgenic mice is represented in (Figure 1). All the mice were male (*n* = 10 per group). The animals were randomly divided into four groups. Control group: Physiological saline was intragastrically administered in C57BL/6 mice within the control group. APP/PS1 group: The mice in the APP/PS1 model group were intragastrically administered using physiological saline. APP/PS1+Q group: Quercetin (100 mg/kg) was administered in APP/PS1 model mice. APP/PS1+DON group: DON was administered intragastrically (0.5 mg/kg). The animals were treated once every day for 6 months (Figure 2).

### 2.3. Y Maze Spontaneous Alternation Test (YM-T)

YM-T is an effective method to measure the learning and memory of experimental animals depending on their nature to explore unfamiliar environments [27]. The direction of the previous exploration should be remembered every time the direction changes. During the training period, each mouse was kept in the center of the device. The number of times each mouse moved in and out of the three arms within 8 min freely was recorded. It should be noted that over one-third of the mouse's body must enter the arm to become a valid record. Spontaneous alternation in the Y maze is determined using the following formula: number of alternations (%) = number of alternations/(total number of arms − 2).

### 2.4. Morris Water Maze (MWM)

The MWM test helped evaluate the changes in spatial learning and memory functions of mice within each group. The MWM comprises a circular pool (diameter 120 cm, height 60 cm) with opaque water (23 ± 1°C) made by adding titanium dioxide pigment and an escape platform (diameter 6 cm). The platform is immersed in the midpoint of a quadrant 1 cm below the water's surface. Mice were brought to the laboratory for 1 h before undergoing cognitive assessment with MWM. Each mouse received training twice daily for 6 days. The latency to escape from the water maze was determined for each trial. If the mouse failed to find the platform within 60 s, it was guided to find it and stay there for 15 s. On Day 7, spatial exploration experiments were performed by removing the platform and allowing each mouse to swim freely for 60 s. The distance and time of swimming within the target quadrant and the number of crossings across the platform were measured. All the data were recorded with video tracking software (WMT-100, China).

### 2.5. Hematoxylin-Eosin (HE) Staining and Nissl Staining

Histological examination was conducted using HE and Nissl staining. The embedded brain tissue was cut into 4 *μ*m sections. The sections were deparaffinized with xylene, dehydrated using different concentrations of ethanol gradients, and stained. Then, xylene was added to the sections to make them transparent and fixed using neutral gum. Morphological changes were observed with computer-assisted light microscopy (CI-L, Nikon, Japan) while measuring and analyzing cell counts using Image-Pro Plus.

### 2.6. Immunofluorescence Staining

The sections were blocked using 5% goat serum for 30 min and incubated overnight with anti-A*β* antibody (mouse monoclonal; 1 : 100, NBP2-13075SS) at 4°C. The sections were washed with PBS and incubated with Alexa Fluor 488- or Alex Fluor 594-conjugated secondary antibodies for 2 h. The images were observed under a fluorescence microscope. After deparaffinization, the tissue sections were washed for 5 min/three times, stained using DIAP for 8 min, and incubated with 3% thioflavin at room temperature for 8 min. Then, they were rewashed using 80% alcohol for 10 s/two times, rinsed using pure water, sealed, and observed under a fluorescence microscope.

### 2.7. ELISA

The mice's blood was centrifuged, and the supernatant was obtained for biochemical assays. We measured the hippocampal ACh levels and AChE activity based on the manufacturer's instructions. Malondialdehyde (MDA) levels, SOD, ROS, and reduced forms in the hippocampus were measured. The GSH-Px activity and other indicators were detected. Hippocampal ACh levels and AChE activity, hippocampal MDA levels, SOD, ROS, and reduced GSH-Px activity were determined with a commercial kit (Enzyme Link, Shanghai, China) following the manufacturer's instructions.

### 2.8. Western Blot

Treated hippocampal tissues and cells were homogenized using RIPA lysis buffer comprising 1% protease inhibitors, followed by centrifuging at 12,000 g for 15 min at 4°C. The supernatant was obtained, and the BCA method helped determine the protein concentration. After SDS-PAGE, proteins were blotted onto the PVDF membrane. The membrane was incubated in 5% skim milk for 1 h using primary antibodies overnight at 4°C. After washing thrice in TBST, the membrane was incubated with HRP secondary antibody for 1 h at room temperature. The ECL method helped detect protein, and the band intensity was quantified using the ImageJ software.

### 2.9. Molecular Docking Analysis of Quercetin

The PDB database (https://www1.rcsb.org/) helped find and retrieve the molecular structure files of the target proteins, namely, Keap1, NQO1, HO-1, Casspase-3, Bcl-2, and Bax. PyMOL 2.3.0 software helped delete water molecules and the original ligands on the downloaded target protein. The PubChem database (https://pubchem.ncbi.nlm.nih.gov/) helped download the quercetin small molecule structure file. Chem3D software was used to perform molecular mechanics determinations on the optimal conformation of small molecules and obtain the optimal conformation with minimized energy. AutoDockTools 1.5.6 was used to hydrogenate the preprocessed target protein molecules. The tool also hydrogenated the small molecules with the optimal conformation procured from molecular mechanics optimization and determined the twistable bonds. The POCASA protein activity pocket online prediction tool helped predict the protein activity pocket while setting the docking range in the predicted active pocket and saving the docking range information for formal docking. AutoDock Vina v.1.2.0 software helped perform molecular simulation target protein and small molecule docking. The Lamarckian genetic algorithm is used for docking. The docking method is semiflexible, with exhaustiveness set to eight and the maximum output conformations set to nine.

### 2.10. Statistical Analysis

The results were represented as mean ± SD and analyzed using one-way analysis of variance (ANOVA). The differences of *p* < 0.05 were considered significant. The statistical tests for each experiment have been depicted in the relevant figure legends, while data analysis was performed using the GraphPad Prism Software 8.0.

## 3. Results

### 3.1. Quercetin Treatment Improves Learning and Memory Deficits in APP/PS1 Mice

**Figure 3:**
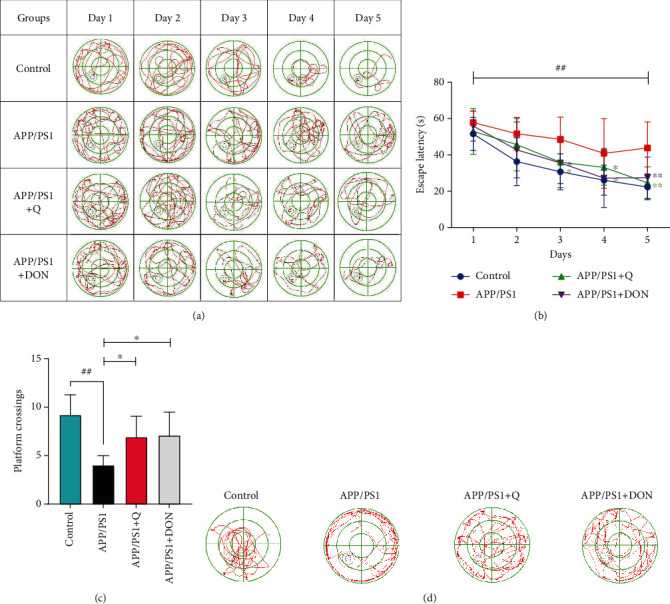
(a) Changes within the average escape latency of mice searching for the hidden platform during five consecutive training days. (b) Mean escape latency. (c) Swimming traces of mice across different groups during the hidden platform test on Day 7. (d) The representative swimming traces of four mice groups during the spatial exploration test. The large circle represents the water maze pool, and the small circle depicts the platform. The data are expressed as mean ± SD; *n* = 10. Compared with the control group, ^#^*p* < 0.5, ^##^*p* < 0.01; compared with the APP/PS1 group, ^∗^*p* < 0.5, ^∗∗^*p* < 0.01 illustrated statistical significance when ^∗^*p* < 0.5.

The mice were tested using MWM and Y maze experiments to verify whether quercetin could enhance memory impairment in APP/PS1 mice. This test evaluates spatial learning and memory function. The MWM results indicated that the model group mice revealed a longer escape latency than the normal group (*p* < 0.05). Therefore, the model group mice had learning and memory impairments (Figures 3(a) and 3(b)). Compared with the model group, the escape latency postquercetin treatment was significantly reduced (*p* < 0.05, *p* < 0.01). A space exploration experimental test was conducted on the seventh day of the water maze. Mice treated with quercetin and DON required less time to reach the hidden platform, showing reduced escape latencies. In addition, the model group crossed the platform fewer times than the normal group (*p* < 0.01). Quercetin and DON treatment increased platform crossings compared to model group animals (Figure 3(c); *p* < 0.01). Each group of mice is swimming in track in the space exploration experiment (Figure 3(d)). Thus, the MWM assay indicated that quercetin treatment enhanced learning and memory among mice.

**Figure 4:**
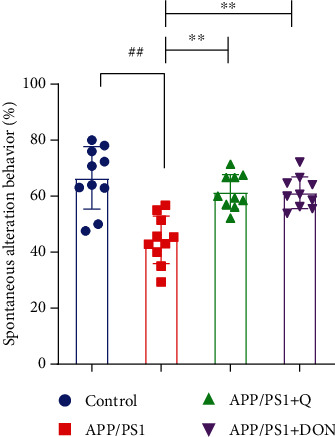
Quercetin treatment enhanced memory in APP/PS1 mice. The data are expressed as mean ± SD. ^##^*p* < 0.01 compared with mice within the normal control group; ^∗∗^*p* < 0.01 compared with mice within the APP/PS1 model group.

The Y maze experiment results showed that APP/PS1 mice had more enhanced spatial memory than normal controls, confirmed by a significant decline in substitution behaviors (*p* < 0.01) (Figure 4). Quercetin treatment significantly reversed these changes, improving spatial memory in APP/PS1 mice.

### 3.2. Quercetin Treatment Reduces Neuronal Damage in APP/PS1 Mice

**Figure 5:**
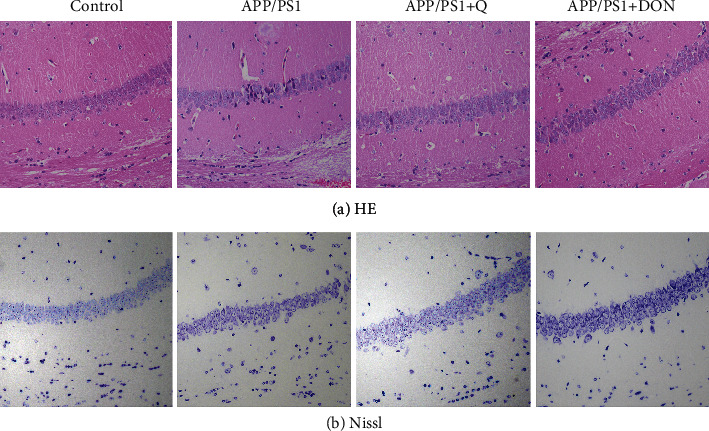
HE and Nissl staining of the hippocampal CA1 region slices (× 200, *n* = 3).

We observed pathological changes using HE and Nissl staining to determine the quercetin treatment effect on neuronal damage in APP/PS1 mice. HE staining revealed that the hippocampal neurons of control mice were regularly arranged, with precise edges and nuclei. In contrast, the hippocampal neurons of APP/PS1 mice were irregularly arranged, possessing blurred structures and shrunken nuclei (Figure 5(a)). Many Nissl-positive bodies were present in the hippocampus of mice within the normal group. The number of Nissl-positive bodies in the hippocampus of APP/PS1 mice was significantly reduced than those in the normal group. APP/PS1 mice treated with quercetin or DON demonstrated a significant rise in the number of Nissl-positive bodies within the hippocampal CA1 region compared with APP/PS1 mice (Figure 5(b)). Thus, quercetin had a protective effect on neuronal damage in APP/PS1 mice.

### 3.3. Immunofluorescence Staining to Detect the Quercetin Inhibited A*β* Production

**Figure 6:**
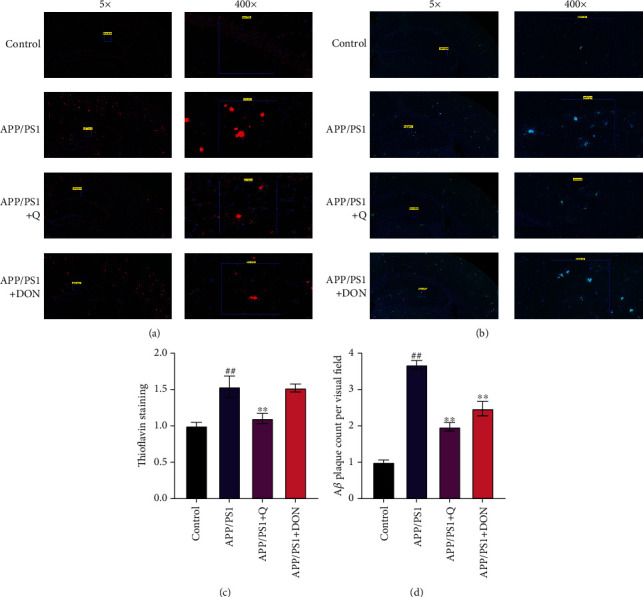
Quercetin treatment decreases senile plaque deposition among APP/PS1 mice. (a) A*β* immunofluorescence labeling reveals A*β* plaques within the mouse cerebral cortex and hippocampus. (b) Thioflavin staining helped observe A*β* deposition inside the cortex and hippocampus of the mouse brain. (c) Quantifying A*β* plaques in the brain of C57BL/6 mice using immunofluorescence. (d) Determining the concentration of A*β* plaques in the brain of C57BL/6 mice using thioflavin staining. Compared with the control group, ^##^*p* < 0.01; compared with the APP/PS1 group, ^∗∗^*p* < 0.01.

A*β* deposition was detected by immunofluorescence staining to understand the mechanism by which quercetin enhances the learning and memory abilities of mice. The results indicated that the amount of A*β* in the mouse cortex and hippocampus was significantly decreased compared with the normal control group. Neuronal damage and neuronal damage in the hippocampus and cortex were observed when APP/PS1 mice were treated with quercetin and DON. Amyloid plaques were significantly reduced (Figure 6(a)), and quercetin alleviated cognitive impairment by inhibiting A*β* production.

### 3.4. Western Blot Validation of Quercetin Regulation of the Keap1/Nrf2/HO-1 Pathway

**Figure 7:**
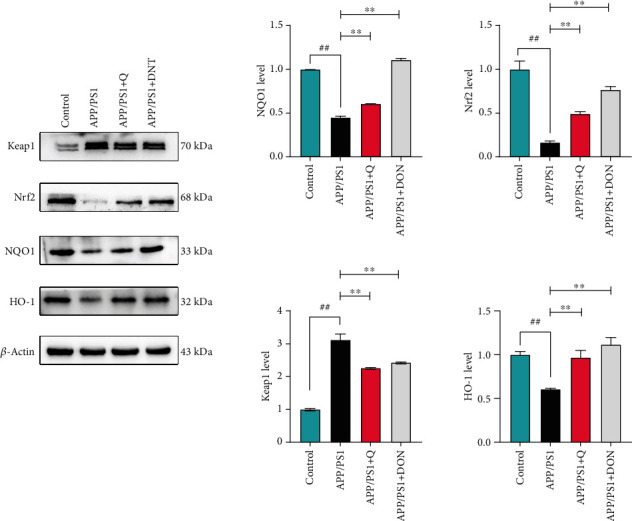
Quercetin treatment enhanced the cognitive ability of APP/PS1 mice. The data is represented as an average of ± SD. ^##^Compared with the normal control group mice, *p* < 0.01. ^∗∗^Compared with the APP/PS1 model group mice, *p* < 0.01.

In order to investigate the effect of quercetin on oxidative stress in APP/PS1 transgenic mice, the changes of oxidative stress index and related proteins of Keap1/Nrf2/HO-1 signaling pathway in hippocampus were analyzed. As shown in Figure 7, the expression of NQO1, Nrf2, and HO-1 in the APP/PS1 model group was significantly decreased (*p* < 0.01) compared with the control group, while Keap1 was significantly elevated (*p* < 0.01). Compared with the APP/PS1 model group, Nrf2 and HO-1 expressions were significantly increased (*p* < 0.01) after feeding DON and quercetin, while Keap1 was significantly reduced (*p* < 0.01). Therefore, DON and quercetin can control the expression of Keap1, NQO1, Nrf2, and HO-1.

### 3.5. Quercetin Inhibits the Expression of Apoptosis-Related Proteins in Neural Cells

**Figure 8:**
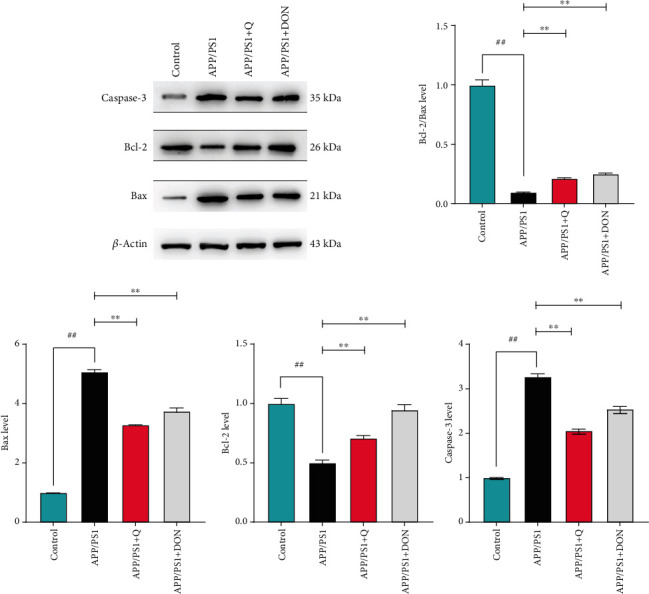
The effect of quercetin on apoptosis proteins. An average of ± SD represents the data. ^##^Compared with the normal control group mice, *p* < 0.01. ^∗∗^Compared with the APP/PS1 model group mice, *p* < 0.01.

To investigate the effect of quercetin treatment on apoptosis events in APP-PS1 transgenic mice, apoptosis levels of hippocampal cells were assessed by quantitative analysis of apoptosis markers caspase-3, Bax, and Bcl-2. Western blot analysis was performed to assess the caspase-3, Bax, and Bcl-2 levels. As shown in Figure 8, Bax and caspase-3 levels in the APP/PS1 group significantly increased compared with the control group, while Bcl-2 levels decreased. After the quercetin treatment group, the protein expression levels were reversed, indicating that quercetin treatment impacted A*β*. The induced apoptosis of mouse brain cells showed antiapoptotic behavior.

### 3.6. Quercetin Inhibits Oxidative Stress Affecting Cholinergic Function in APP/PS1 Mice

**Figure 9:**
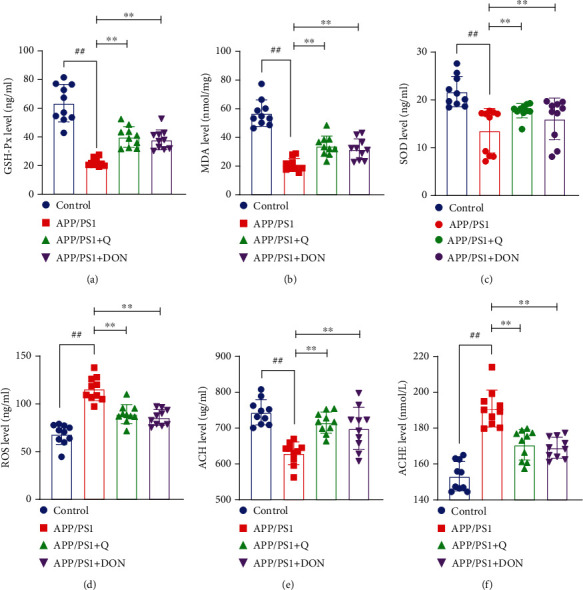
Quercetin treatment effect on serum antioxidant proteins and enzymes in mice. (a) Mouse serum GSH-Px levels, (b) mouse serum MDA levels, (c) mouse serum SOD levels, (d) mouse serum ROS levels, (e) mouse serum ACh levels, and (f) mouse serum AChE levels. The results are represented as mean ± standard deviation (*n* = 10). ^##^Compared with the normal control group mice group, *p* < 0.01. ^∗∗^Compared with the APP/PS1 model group mice, *p* < 0.01.

To explore the effects of quercetin on oxidative damage and cholinergic dysfunction in APP/PS1 mice, the changes in the oxidative stress and cholinesterase in serum and hippocampus were detected. It was observed that the APP/PS1 model group had significantly reduced serum GSH-Px, MDA, and SOD (*p* < 0.01) levels than the normal control group to detect ROS and antioxidant enzyme activities. Compared with the APP/PS1 model group, quercetin treatment inhibited the decline in GSH-Px, MDA, and SOD levels (*p* < 0.01). A significant enhancement in total ROS activity was observed in the serum of APP/PS1 model group mice (*p* < 0.01). Moreover, quercetin treatment significantly decreased ROS levels than in the model group (*p* < 0.01) (Figures 9(a), 9(b), 9(c), and 9(d)). As shown in Figures 9(e) and 9(f), ACh activity levels in the hippocampus of APP/PS1 model mice decreased (*p* < 0.01) and increased (*p* < 0.01). Quercetin treatment enhanced ACh activity (*p* < 0.01) while significantly reversing the abnormal rise in AChE level activity (*p* < 0.01).

Figures 9(e) and 9(f) revealed that the Ach activity levels in the hippocampus of APP/PS1 model mice were reduced (*p* < 0.01) and enhanced (*p* < 0.01). Quercetin treatment elevated ACh activity (*p* < 0.01) while significantly reversing the abnormal rise in AChE level activity (*p* < 0.01).

### 3.7. Molecular Docking Analysis of Quercetin

**Figure 10:**
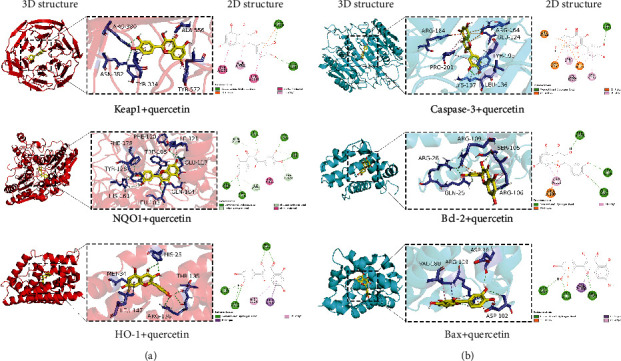
Molecular docking analysis of quercetin with active protein target sites. (a) Three-dimensional (3D) and two-dimensional (2D) structural representations of Keap1, NQO1, and HO-1 interactions. (b) The three-dimensional (3D) and two-dimensional (2D) structural representations of the interactions involving Bax, Bcl-2, and caspase-3.

In order to further explain the degree of correlation between quercetin and each target, the molecular docking analysis of the key target was carried out. The binding energy was less than −5 kcal/mol, with excellent binding performance. Moreover, the binding effect was strong when it was < −7 kcal/mol. Quercetin exhibits significant binding interactions with protein targets, including Keap1, NQO1, HO-1, Bax, Bcl-2, and caspase-3 (Table 1). The docking situation of each group was visualized and analyzed separately. The docking results were observed using PyMOL 23.0 software and Discovery Studio 2020. Figures 10(a) and 10(b) represent the three-dimensional and two-dimensional structural arrangements of quercetin with various proteins. The binding energy and docking situation graph reveals that the binding affinity between the six protein sites and quercetin is good, effectively docking on the binding sites of different protein targets. The binding energy of the protein NQO1 group was the lowest. Therefore, it was speculated that the binding between quercetin small molecules and protein NQO1 was the best among the five small molecules.

## 4. Discussion

AD pathogenesis is not fully understood, but increasing evidence focuses on ROS-mediated oxidative stress. Currently, there are no fully effective drugs to treat AD. Quercetin, a naturally occurring flavonoid extracted from various plants, vegetables, and fruits, can potentially treat and prevent neurodegenerative diseases, showing various pharmacological effects [28]. It is often used as a free radical scavenger, powerful antioxidant. Quercetin can improve learning and neurotrophic effects while inhibiting amyloid production. Additionally, quercetin can improve memory and cognitive functions in AD patients, revealing good therapeutic effects [29]. The present study used mainly an APP/PS1 double transgenic mouse model, which is useful for studying the neuropathology mechanisms of AD and evaluating the therapeutic effects of various anti-AD drugs [30]. Through the MWM and YM-T experiments, we found that quercetin can improve the learning disability and memory ability of the model mice.

AD-induced behavioral dysfunction could be related to structural and functional impairments within specific brain regions. The hippocampus and cortex are vulnerable areas affected by A*β* [31]. Histopathological techniques helped highlight changes in plaque structures and cell morphology. The results indicated that APP/PS1 mice demonstrated massive A*β* deposition and neuronal damage within the hippocampus and cortex, significantly reducing Nissl bodies. These A*β* plaques induce oxidative stress and mitochondrial dysfunction, producing neuronal cytotoxicity [7]. A*β* plaques were significantly reduced in this study in the quercetin-treated group than in the DON-treated group. Thus, quercetin treatment helped attenuate neuronal damage and A*β* deposition.

Oxidative stress is closely associated with the neurodegenerative pathogenesis of AD. A*β* aggregate accumulation may aggravate ROS production and impair synaptic activity and cognitive function [32]. The Keap1/Nrf2/HO-1 signaling pathway has been an endogenous module of the redox reaction. The pathway inhibits apoptosis and oxidative stress by controlling mitochondrial function to decrease transcription factors during the redox reaction [33]. Under normal conditions, Nrf2 and Keap1 are combined in the cytoplasm and are ubiquitinated and degraded. Nrf2 dissociates from Keap1 and enters the nucleus after oxidative stress stimulation and various antioxidants [34]. Combined with the nuclear antioxidant response element, it initiates the downstream factors HO-1 and NQO1 expression to remove ROS. Therefore, the Keap1/Nrf2/HO-1 signaling axis protects against oxidative stress damage [11]. Decreased transcription factor Nrf2 expression was observed in AD brains, and Nrf2 induction enhanced cognitive impairment in AD model mice. We detected Nrf2 protein expression to assess whether quercetin activates HO-1 and NQO1 by enhancing Nrf2 expression, protecting AD model mice from oxidative stress. The results indicated that quercetin treatment decreased Keap1 expression in the hippocampus of model mice and improved the antioxidant protein Nrf2 expression. Then, the treatment increased the expression of HO-1 and upregulated the antioxidant protein NQO1 expression. These findings support quercetin in treating AD by inhibiting oxidative stress. In addition, quercetin can inhibit lipid peroxidation progression and improve the body's antioxidant defense system. Under normal physiological conditions, several enzymes, such as ROS, GSH-Px, MDA, and SOD, enhance the free radical effects on intracellular processes and organelle protection [35]. The experimental results indicated that the total antioxidant activity of the APP/PS1+Q group increased compared with the APP/PS1 group. This prevented oxidation processes while protecting the body from oxidative damage. SOD is the body's main antioxidant enzyme scavenging free radicals, reflecting the body's ability to scavenge free radicals and assess the oxidative damage degree to organs. MDA is a product of free radical-induced lipid peroxidation [36]. GSH-Px is an effective free radical scavenger inhibiting ROS production and is the first defense line against nonenzymatic oxidative stress [37]. This study evaluated various oxidative stress parameters. The results indicated that the quercetin treatment group significantly decreased ROS content and increased MDA, SOD, and GSH-Px content. Therefore, quercetin can eliminate A*β*-induced ROS, elevate the antioxidant enzyme activity, and decrease cellular oxidative stress damage by activating the Nrf2 signaling pathway.

In addition, many studies have demonstrated that Nrf2 also plays an essential role in oxidative stress–induced apoptosis [38, 39]. Massive neuronal loss is a prominent feature of AD patients, wherein abnormal cell apoptosis becomes vital in AD pathology [35]. Apoptosis occurrence in the cortical center and hippocampus affects memory and learning [40]. As proapoptotic proteins in the Bcl-2 family, Bax and caspase-3 are involved in the activation of death proteases of the cysteine protease family and are linked with cell apoptosis [41]. Proapoptotic substance overexpression can significantly accelerate cell apoptosis. In this study, western blot analysis helped determine the expression of Bcl-2, Bax, and caspase-3 to decipher the protective effect of quercetin on A*β*-induced apoptosis. Apoptosis was significantly enhanced in APP/PS1-induced mice. However, quercetin treatment significantly decreased the apoptotic protein expression in the hippocampus of brain tissue. This study demonstrated the protective effect of quercetin on A*β*-induced apoptosis.

Quercetin displays significant AChE inhibitory activity. The cholinergic system is crucial in neurobiological processes, such as memory and cognition. Thus, degeneration of hippocampal cholinergic neurons may induce the loss of learning ability and memory related to neurodegenerative diseases such as AD, one of the iconic features [42]. ACh is a key neurotransmitter in cholinergic signaling associated with memory and cognitive processes [43]. Moreover, AChE is a key enzyme in neurotransmission that hydrolyzes ACh into acetate and choline and terminates ACh-mediated synaptic transmission inside the central nervous system with significant catalytic efficiency [44]. Here, hippocampal AChE activity in APP/PS1 model mice was significantly elevated, indicating cholinergic dysfunction. However, quercetin treatment significantly reduced AChE activity.

## 5. Conclusions

**Figure 11:**
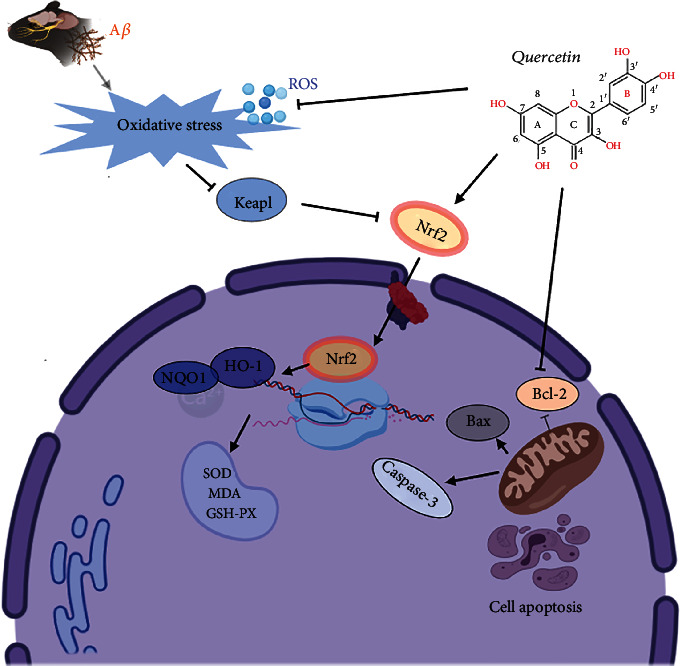
The potential quercetin mechanism enhancing cognitive impairment in AD mice.

Therefore, quercetin improved learning and memory impairment, inhibited the amyloidogenic process, suppressed oxidative stress, enhanced AChE activity via the Keap1/Nrf2/HO-1 pathway, and prevented cell apoptosis in APP/PS1 transgenic mice (Figure 11). The study results may provide a molecular basis to clinically apply quercetin while treating AD.

## Figures and Tables

**Table 1:** Molecular docking results of quercetin with Keap1, NQO1, HO-1, Bcl-2, Bax, and caspase-3 target proteins.

**Target proteins**	**Binding energy (kcal. mol** ^**−1**^ **)**	**Center (** **X**, **Y**, **Z)**	**Docking size (** **X**, **Y**, **Z)**	**Interactions**
Keap1	−7.9	center_*x* = −42.132 center_*y* = 23.535 center_*z* = 60.847	size_*x* = 17.67 size_*y* = 17.67 size_*z* = 24.73	ARG (A:380), ASN (A:382), ALA (A:556), TYR (A:334), TYR (A:572)
NQO1	−9.1	center_*x* = 11.884 center_*y* = −0.31 center_*z* = 10.373	size_*x* = 68.0 size_*y* = 76.0 size_*z* = 62.0	GLN (A:104), GIU (C:117), PHE (C:120), PHE (C:178), HIS (A:161), TYR (C:128), TRP (A:105)
HO-1	−7.5	center_*x* = 16.831 center_*y* = −20.475 center_*z* = 10.373	size_*x* = 60.0 size_*y* = 44.0 size_*z* = 52.0	ARG (A:136), THR (A:135), MET (A:34), LEU (A:147), HIS (A:25)
Caspase-3	−7.7	center_*x* = 4.054 center_*y* = 0.026 center_*z* = 6.748	size_*x* = 15.0 size_*y* = 20.25 size_*z* = 15.0	ARG (A:164), ARG (B:164), GLU (B:124), PRO (A:201), LEU (B:136), LYS (B:137), TYR (B:195)
Bcl-2	−7.0	center_*x* = −6.849 center_*y* = −1.228 center_*z* = 40.163	size_*x* = 47.25 size_*y* = 45.0 size_*z* = 47.25	SER (A:105), ARG (A:26), ARG (A:106), ARG (A:109), GLN (A:25)
Bax	−7.2	center_*x* = −8.17 center_*y* = −0.333 center_*z* = 10.373	size_*x* = 46.0 size_*y* = 54.0 size_*z* = 40.0	ARG (A:164), ARG (B:164), GLU (B:124), PRO (A:201), LEU (B:136), LYS (B:137), TYR (B:195)

## Data Availability

The data used to support the findings of this study are available from the corresponding author upon request.
